# Role of Apparent Diffusion Coefficient in Evaluating Degeneration of the Intervertebral Disc: A Narrative Review

**DOI:** 10.7759/cureus.43340

**Published:** 2023-08-11

**Authors:** Anshul Sood, Gaurav V Mishra, Manasa Suryadevara, Pratap Parihar, Shreya Khandelwal, Nishtha Manuja, Keyur Saboo, Sheetal S Shelar, Abhinav Ahuja, Nitish Batra

**Affiliations:** 1 Radiodiagnosis, Datta Meghe Institute of Higher Education and Research, Jawaharlal Nehru Medical College, Wardha, IND; 2 Internal Medicine, Datta Meghe Institute of Higher Education and Research, Jawaharlal Nehru Medical College, Wardha, IND

**Keywords:** ivdd, mri, diffusion, degenerative disc, t1p, t2*, dti, fa, dwi, adc

## Abstract

Degeneration of the lumbar intervertebral disc is the most common cause of lower back pain. It is directly related to daily activities, mechanical stress, and other biological factors. We use imaging modalities to assess the degree of disc degeneration, out of which magnetic resonance imaging (MRI) is the most popular non-invasive modality. It is believed that early changes in disc degeneration are due to the biochemical events in the disc and can be evaluated by sequences in MRI involving the diffusion of water molecules. The apparent diffusion coefficient (ADC) is one such sequence that captures the signals based on the diffusion of water molecules. Ten articles were chosen from PubMed and Google Scholar using the MeSH terms 'lumbar spine degeneration' and 'apparent diffusion coefficient'. This review article has summarized various studies intending to gain a better understanding of the biochemical events leading to the development of disc degeneration. This study has also gathered the role of various sequences in MRI that can quantitatively assess disc degeneration.

## Introduction and background

The most common cause of lower back pain is intervertebral disc degeneration (IVDD) [1]. The intervertebral disc (IVD) acts as a cushion between two adjoining vertebrae. It comprises three major components: central nucleus pulposus (NP), surrounded by outer annulus fibrosus and cartilaginous end plates, which anchor the disc and the vertebral body [2]. The NP comprises 66-86% water, and proteoglycans and collagen occupy the remainder percentage, predominantly type 2 collagen [2]. With advancing age and other risk factors like heavy weightlifting, degeneration of the disc occurs, starting with a sequence of biochemical events including degradation of the extracellular matrix and breakdown of the large proteoglycan gel aggregates, leading to morphologic changes and alteration in the biomechanical properties [3]. Magnetic resonance imaging (MRI) is considered the best non-invasive method for characterizing IVDD as it can reflect on the disc's structural integrity and macromolecular concentrations [4,5]. A non-invasive way to calculate the diffusion of water within the tissues is by using diffusion-weighted MRI, and an approximation of the free diffusion of unbound water can be measured by the apparent diffusion coefficient (ADC) [6]. This review article has summarized multiple studies aiming to understand better the role of ADC in evaluating the degeneration of the IVD.

## Review

Materials and methods

**Figure 1:**
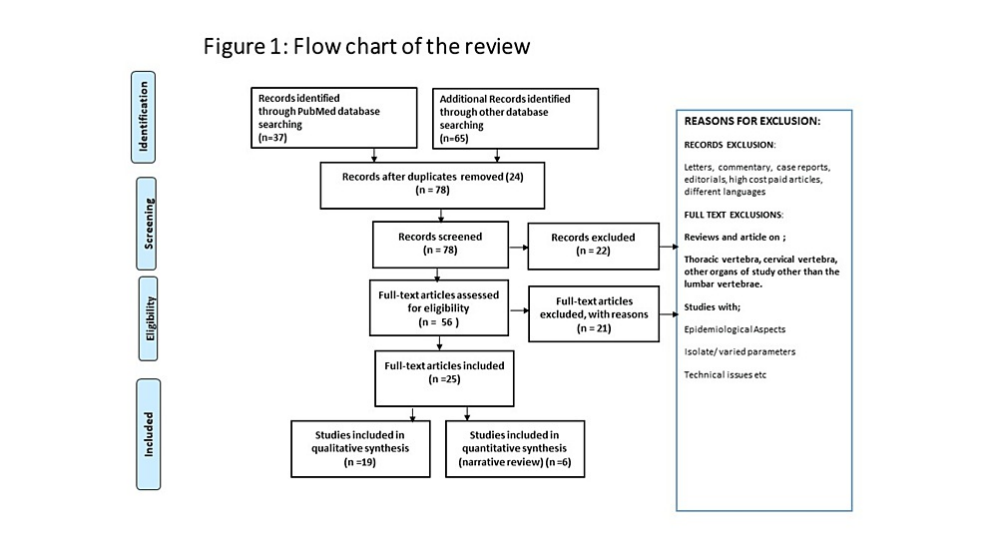
Flow chart of the review.

We searched PubMed and Google Scholar for studies reporting on the ADC and other MRI sequences that can be used in detecting the early changes of the degeneration taking place in the spine. The articles chosen for this narrative review have been searched from accredited sites PubMed and Google Scholar. The keywords used were 'lumbar spine degeneration' and 'apparent diffusion coefficient'. We mainly used articles in the English language that were published in the international literature. The flow chart of the review has been highlighted in Figure 1.

We aimed to provide data on the role of ADC in detecting changes in the disco-vertebral complex.

Review of literature

IVDD is the most common cause of lower back pain [1]. Three major components of the IVD are NP which is the innermost central portion of the disc; annulus fibrosus, which surrounds the NP and lies in the periphery; and cartilaginous end plates, which help in the anchoring with the vertebral bodies [2]. NP comprises water, proteoglycans, and collagen and can be considered a branching of numerous thin, randomly placed collagen fibres interspersed with proteoglycans. The interpenetration of the proteoglycans provides space between the collagen fibres and forms tiny pores, which help move small solutes and water. Proteoglycan concentration decides the pore measurements and the swelling pressure. Proteoglycans are negatively charged and result in high osmotic pressure. Therefore, the amount of water a disc can attract is directly proportional to the number of proteoglycans [6].

The nutrition to the disc is provided either by diffusion through the cartilaginous end plate of the vertebral bodies or from the surrounding capillaries through the annulus fibrosus, and the waste product of metabolism is excreted through the reverse route [7,8]. There is no vascular supply to the NP throughout life. In the second decade, the blood supply to the annulus fibrosus is also discontinued [6], making the IVD of the lumbar spine the biggest avascular structure of the body [6-8]. The cells in the disc can be more than 5 mm away from the nearest vessel. So, the metabolites are primarily transported via the matrix of the disc by molecular diffusion in response to the concentration gradients and, to some extent, by the change in the fluid volume of the disc due to activity [6-8]. Diffusion changes are thought to be the earliest marker of disc degeneration [6].

In human beings, the process of degeneration of the disc starts in the second decade of life when the person is approximately 16-20 years of age initiated due to mechanical factors, physiological degeneration and high load stress on the spine, and various other factors can accelerate the process of degeneration [9,10].

Degeneration of the disc initiates through biochemical changes causing metabolism imbalance in the NP, mainly involving collagen damage and a gradual loss of proteoglycan. These changes are not accompanied by significant structural changes and are thought to be the earliest markers of degeneration [8]. Also, under higher pressure, the cells of an IVD in the lower lumbar levels regulate the metabolic activities in various ways, causing an increase in the production of pro-inflammatory cytokines such as matrix metalloproteinases (MMPs), interleukin-1 (IL-1) and aggrecans [11]. These enzymes cause degradation of the matrix, destroying hydrophilic glycosaminoglycans within the NP (particularly aggrecan), leading to the accumulation of cleaved matrix fragments like fibronectin [12] which further stimulates the catabolic responses by binding to the cell surface receptors [13]. Due to these biochemical changes, there is a decrease in the osmotic pressure leading to water extraction from the disc [14].

Women are at an increased risk of developing severe disc degeneration when compared to men of the same age group in old age. This discrepancy is most likely because of the estrogen deficiency causing accelerated disc degeneration, either by directly stimulating the effect on the cells of IVD or indirectly affecting vertebral marrow perfusion [15].

The two most common imaging methods to evaluate reactive bony changes and disc morphology are conventional computed tomography and radiography [1,3]. The earliest changes of disc degeneration involve biochemical events before morphologic changes, including degradation of extracellular matrix and breakdown of large aggregating proteoglycan gels, and hence are generally undetected by the two methods mentioned above [3].

MRI is considered the best non-invasive method to check for structural integrity and macromolecular concentrations in the IVD [4,5]. T1-weighted (T1WI) and T2-weighted (T2WI) MRI sequences provide the anatomical details of the soft tissues, along with water, and fat content. These sequences mainly focus on the organizational changes in the disc and zygapophyseal joints, the extent and location of the disc material displacement, reactive vertebral bone marrow changes, the degree of stenosis, and the extent and location of compression of nerve roots [16]. It can also provide information about the Modic changes, schmorl nodules and protrusion or extrusion of the IVD [17].

Based on the signal strength of the IVD in T2WI, the Pfirrmann grading system has been established to classify IVDD. The Pfirrmann grading system is based on four elements of the sagittal T2WI MRI images, i.e., the signal strength of the NP, the height of the lumbar disc nucleus, the structure of the nucleus and the boundary between the annulus fibrosus and NP [18,19]. This grading system helps diagnose disc degeneration [20]; however, it is useless in assessing early disc degeneration, whose key role is identifying ultra-structural alterations [21]. Also, no experimental evidence supports the hypothesis that two discs, classified under the same degenerative category based on visual appearance, are functionally similar in their degenerative progression [22]. Therefore, other sequences involving the diffusion of water molecules can be used to detect early changes in the degenerative disc [9].

Diffusion consists of the Brownian (random) translational motion of protons. Diffusion-weighted MRI can measure the movement of water molecules within the tissues, and the free diffusion of unbound water can be estimated using the ADC [6]. The movement and location of water molecules within the tissue affect the ADC values and are dependent on the motion of water molecules within the cells and across the cells, blood perfusion of tissues and movement of extracellular water molecules. When there is no blood supply to the NP and the inner layer of the annulus fibrosus, ADC values are determined by the diffusion of extracellular water [23]. With an increase in the diffusion of water molecules, there is an increase in the ADC values, a decrease in the DWI values and faster signal attenuation [24].

A study done by Niu G et al. [25] involved 60 individuals, and the classification of all the discs was done based on the degree of disc extension beyond the interspace (DEBIT) into non-bulging/herniation disc group, bulging disc group and herniation disc group (extrusion or protrusion). Classification of discs for the evaluation of degeneration was based on (i) reduction of the signal intensity on T2WI, (ii) loss of disc height and (iii) loss of distinctness of annulus fibrosus with NP. Discs with a minimum of two of these findings were termed as severely degenerated, with only one as mildly degenerated, and discs with none as non-degenerated discs. It was found that most of the non-bulging/herniated discs were cephalic lumbar vertebral discs, and mostly bulging and herniated discs were present at the caudal lumbar level. A weak inverse correlation (P<0.01) was established between the ADC values of NP and lumbar disc level. Also, the ADC values of the non-bulging/herniated discs were significantly higher than those of the degenerated and bulging discs, suggesting a statistically notable difference (P<0.05).

A study done by Yu Hon et al. [22] calculated the ADC by drawing a region of interest (ROI) to determine IVD-based ADC values using two different approaches: (i) after pixel-by-pixel calculation of ADC value in the ROI, the mean ADC value was obtained; (ii) mean signal intensities of two b values were calculated within the ROI and then a single ADC value was calculated. A mid-sagittal ROI was also separately utilized to determine ROI-averaged and pixel-mean ADC values. On comparing ADC values at the mid-sagittal ROI to that of IVD ROI, it was found that ADC values are higher in the former because it consisted only of NP while the latter consisted of NP and a more prominent presence of annulus fibrosus, which have lower ADC values when compared to NP [26]. Also, it was found that pixel-by-pixel calculation of the ADC value was consistently lower than the ADC values calculated by using mean signal intensities of two b values, even though the ADC values are from the same IVD. This study suggests that the change in ADC value in the same IVD can be noted and it is dependent on the way ADC values are calculated.

Shen Si et al. [9] compared DWI, diffusion tensor imaging (DTI) and T2* mapping of the IVD in the lumbar level in 40 young, healthy adults without having lower back pain. GE-Functool software was used to generate ADC from DWI, which reflects the isotropic diffusion, fractional anisotropy (FA) from DTI, which reflects the proportion of anisotropic diffusion, and T2* pseudo colour images, which is based on T2 mapping with a clearer imaging contrast and a higher signal-to-noise ratio (SNR) [27,28]. The central regions of the NPs were split equally into five parts, and ROIs were laid down from anterior to posterior. Statistical analysis showed significant differences in ADC, FA and T2* values among different ROIs in the same IVD. The mean values of T2* and ADC were found to be highest in the centre and gradually declined towards the edges (P<0.05); the value of FA was lower at the centre and gradually increased towards the periphery. This study found that DWI, T2* and DTI mapping could act as a non-invasive, sensitive method for quantitatively characterizing the biochemical status of NP. The increase in the ADC, T2*, and FA values of the NP could be imaging markers for early lumbar disc degeneration.

A study by Zhang W et al. [29] showed a negative correlation of the ADC values with degeneration of the disc at all spinal levels. They proposed that the ADC values depend on the severity of degeneration and that ADC is maximally affected by disc degeneration at the L5/S1 disc level.

Water content in the cells, mechanical properties and osmotic pressure are some indicators closely related to prostaglandins and are closely related to T1P values [30]. It is reported by Filippi et al. [31] that T1p values can sensitively reflect changes in the prostaglandins of the IVD.

In a study done by Cui Y et al. [24] involving 90 individuals, a relationship between the T1p values of the NP and annulus fibrosus and the composition of the IVD was established and was found to be statistically significant (P<0.05). T1p values were represented by using T1p pseudo-colour images using colour gradations. NP appeared yellowish green in the outer peripheral region and orange, representing a high-value region in the core, as opposed to the uniform signal intensity of the disc in T2WI. The decrease in the T2WI signals gradually showed a uniform blue colour. This study found that T1p values of the degenerated discs were lower than non-degenerated, healthy discs.

A study by Belykh E et al. [16] studied 452 lumbar vertebral segments and found an association between the increased ADC values and Modic type 1 changes, which states an increase in the diffusion in this area compared to the end plates of the vertebra not having Modic changes or having type 2 Modic changes. This increase in diffusion shows either the infectious [32] or edematous [33] nature of the pathologic process. Ten percent of segments with Modic type 2 changes were found to have high mean ADC values like those noted in type 1 Modic changes. This highlights the intricacy of the biochemical mechanisms of bone marrow changes in the reactive vertebral body. This study also found that grades 4 and 5 of the degenerated discs having lower ADC values show an increased possibility of developing Modic changes.

Some of the specific uses of ADC maps regarding the disco-vertebral complex include diagnosis of lumbar vertebral chordoma [34], evaluation of bone mineral density of vertebrae [35], or response of myeloma to treatment [36]. 

This study found out that the disc starts to degenerate from the second decade of life and the initial process of degeneration involves the biochemical changes rather than the structural changes. So, by using various sequences like DWI, ADC, DTI, FA, T2*, T1p and T2 mapping, it is possible to quantitatively measure the disc degeneration. A list of the results of the review has been shown in Table 1.

**Table 1:** Population data table. ADC, apparent diffusion coefficient; DWI, diffusion-weighted imaging; DTI, diffusion tensor imaging; FA, fractional anisotropy.

Reference	Number of subjects	MRI sequences used	Conclusion
Niu G et al [25]	28 cases and 32 controls	T2WI and ADC	ADC value decreased with increasing disc degeneration.
Yu Hon et al. [22]	90 healthy volunteers	T2WI and ADC	ADC value might change depending on the technique of calculation.
Shen Si et al. [9]	44 healthy volunteers	T2WI, T2*, DWI, ADC, DTI, FA	Positive correlation of DTI, DWI and T2* to calculate diffusion of water.
Zhang W et al. [29]	109 subjects (60 women and 49 men)	T2WI and ADC	Negative correlation of ADC values with degeneration of the disc at all spinal levels.
Filippi et al. [31]	34 subjects (19 women and 15 men)	T2WI and T1p	T1p values can sensitively reflect the changes in prostaglandins of the intervertebral disc.
Cui Y et al. [24]	60 cases and 30 controls	T2WI, T2 mapping, DWI and T1p	T2WI, T2 mapping, DWI and T1p sequences have different capacities to diagnose degeneration of the lumbar disc and help in increasing diagnostic accuracy.
Belykh E et al. [16]	100 consecutive patients operated for various pathologies in the lumbar spine	T1WI, T2WI and ADC	Grades 4 and 5 of the degenerated discs having lower ADC values show an increased possibility of developing Modic changes.
Kerttula L et al. [6]	18 healthy young volunteers	ADC	Scatter was noted in the ADC values of these volunteers, perhaps due to non-alignment of the slice plane parallel to the disc suggesting anisotropic diffusion of the nucleus pulposus.

## Conclusions

This review paper discussed the role of ADC values in evaluating the changes in early lumbar disc degeneration. This article aimed to analyze the processes that contribute to the development of disc degeneration and to find a quantitative way to estimate the degeneration of the disc. Disc degeneration starts at a very early age and is caused by biochemical changes in the disc. Many systems for evaluating disc degeneration have been proposed, out of which the Pfirrmann classification system is the most widely used. However, it is seen that the two discs having the same grading in the Pfirrmann classification system may have completely different levels of disc degeneration due to biochemical events. It is essential to determine the quantitative method of evaluating the changes in disc degeneration. Many such sequences as DWI (ADC), DTI (FA) and T2* mapping have been put forward, which involve the diffusion of water molecules to determine disc degeneration.
